# Infants’ conceptual representations of meaningful verbal and nonverbal sounds

**DOI:** 10.1371/journal.pone.0233968

**Published:** 2020-06-08

**Authors:** Louah Sirri, Ernesto Guerra, Szilvia Linnert, Eleanor S. Smith, Vincent Reid, Eugenio Parise

**Affiliations:** 1 Department of Education, Manchester Metropolitan University, Manchester, United Kingdom; 2 Department of Psychology, Lancaster University, Lancaster, United Kingdom; 3 Institute of Education and Center for Advanced Research in Education, Universidad de Chile, Santiago, Chile; 4 Department of Experimental Psychology, University of Cambridge, Cambridge, United Kingdom; 5 School of Psychology, University of Waikato, Waikato, New Zealand; University of Portsmouth, UNITED KINGDOM

## Abstract

In adults, words are more effective than sounds at activating conceptual representations. We aimed to replicate these findings and extend them to infants. In a series of experiments using an eye tracker object recognition task, suitable for both adults and infants, participants heard either a word (e.g. cow) or an associated sound (e.g. mooing) followed by an image illustrating a target (e.g. cow) and a distracter (e.g. telephone). The results showed that adults reacted faster when the visual object matched the auditory stimulus and even faster in the word relative to the associated sound condition. Infants, however, did not show a similar pattern of eye-movements: only eighteen-month-olds, but not 9- or 12-month-olds, were equally fast at recognizing the target object in both conditions. Looking times, however, were longer for associated sounds, suggesting that processing sounds elicits greater allocation of attention. Our findings suggest that the advantage of words over associated sounds in activating conceptual representations emerges at a later stage during language development.

## Introduction

Two key features of human cognition are language and conceptual categorization [1]. Developmental studies have shown that during the first years of life, verbal (spoken words)—as opposed to non-verbal meaningless—sounds facilitate conceptual categorization [2, 3]. Conceptual categorization implies constructing an abstract mental representation of a category by grouping different exemplars or objects into clusters based on shared features, such as perceptual, functional, taxonomic or thematic [4]. Consequently, upon hearing the label of an object (e.g. ‘dog’), all category related objects (e.g. exemplars of dogs, and/or associated animals (e.g. cat, sheep) are activated, which in turn, results in faster visual object recognition [5, 6]. Meaningful auditory information, however, originates not only from spoken words (e.g. “dog”), but also from environmental associated sounds (e.g. bark). Although words and associated sounds are both informative and semantically related to their referents (e.g. dog), they fundamentally differ from one another [7]. While associated sounds are based on causal relationships being strictly related to their generating source, words are arbitrarily linked to their referents, have phonological forms that are reproduced by a person, carry an informative intent, and are used to label objects or name a category to which these objects belong [8, 3].

Though both words and associated sounds carry semantic knowledge, research on language processing and object recognition has focused mostly on spoken words, and much less on associated sounds. It remains unclear whether during language development, infants process words and associated sounds similarly, or whether the former has an advantage over the latter. Uncovering what effects words have on developing conceptual representations can contribute to a better understanding of the relation between language and cognition. An important question is whether words and associated sounds activate conceptual representations differently, and consequently, whether recognizing visual information can be modulated by a preceding auditory cue (e.g. word *versus* associated sounds). In adults, a few studies that investigated the semantic organization of words and associated sounds have shown that compared to words, associated sounds enhanced visual object detection (judging whether an object was present on the visual display) [9], especially when the stimulus onset asynchrony (SOA; time from the beginning of the auditory stimulus to the appearance of the image) was short (e.g. 350 ms; 10). Chen & Spence [10]suggested that words access their meanings via lexical representations, whereas associated sounds access faster and more directly their meaning. The findings of a more recent study using the visual world paradigm (VWP), revealed that participants looked faster at the target (e.g. puppy) and longer at its competitor (e.g. bone) compared to other two distractors (e.g. candle and daffodil), suggesting similar graded effects for both associated sounds and words [11]. In addition, the results showed that this graded pattern was more pronounced in the associated sounds condition [11].

On the other hand, when investigating the activation of conceptual representations during object recognition, Lupyan and Thompson-Schill [8]found that words (e.g. ‘dog’) activate conceptual representations more effectively than associated sounds do (e.g. dog bark). In a series of visual identification tasks, adults systematically reacted faster to a target image when primed by a word compared to an associated sound. In another VWP study [6], the results also revealed that sound primes led participants to look more at one category exemplar (e.g. robin) compared to the three others (e.g. three different exemplars of *bird*), most likely the source of the sound, whereas in response to word primes, participants looked equally at the four images. These findings suggest that while words are somehow detached from the perceptual information, sounds are tightly linked to the perceptual details of the generating source [6]. Furthermore, although both words and sounds yielded similar N400 response (an event-related brain potential (ERP) known to reflect semantic processing; [12]), words elicited earlier and larger P1 ERP response, which is related to perceptual categorization [5].

Altogether, Lupyan and colleagues’ work contrasts previous hypotheses that words and sounds are processed similarly [13], or that sounds access their meanings faster than words [10]. They demonstrate that although both words and sounds activate conceptual representations, the representations activated by words are enhanced, facilitating the match to the category exemplars. Unlike associated sounds, labels are abstract symbols “standing for” and referring to objects. They are used by humans to communicate and convey abstract information that is not strictly linked to the ‘here and now’ of an object, whereas associated sounds are mere features of objects [8, 14]. According to Edmiston and Lupyan [6], sounds act as “motivated” cues, and are idiosyncratically linked to their referents, whereas words are decontextualized “unmotivated” cues, and activate conceptual categories abstractly. By taking this stance, we were interested in determining whether differences in processing words and associated sounds occur early in language development. This will contribute to a better understanding of how we form categories, and to the theoretical account stating that ‘words refer to’ [3], rather than being merely ‘associated to’, objects (3, for a review). According to this account, words enable more abstract conceptual representations and are not directly linked to the context or event, therefore enhancing object recognition.

To the best of our knowledge, there are only two developmental studies that investigated whether young children process known words and sounds similarly [15, 16]. In Cummings et al. [15] study, 15-, 20-, and 25-month-old toddlers participated in a looking-while-listening task, during which they viewed pairs of images (e.g. dog–piano) and heard either associated sounds (e.g. dog barking or piano playing) or words. The results showed that across ages, infants were equally fast at recognizing the target object in both word and associated sound conditions. Faster object recognition preceded by words was correlated with infants’ productive skills. In their recent ERP study, Hendrickson et al. [16]investigated the semantic organization of words and associated sounds in the developing brain of 20-month-olds, including three control conditions. Toddlers viewed the target images (e.g. dog) while hearing matching words (e.g. “dog”) or associated sounds (e.g. barking), within-category violations (“cat” or meowing), and between-category violations (e.g. “pen” or scribbling). The ERP results showed that 20-months-olds exhibit different patterns of brain activation in response to words and associated sounds. While between-category violations (e.g. dog–“pen” or scribbling) elicited similar ERP responses across words and associated sounds, within-category violations (e.g. dog–“cat”) for words elicited earlier and greater negativity than for associated sounds (e.g. dog–meowing), suggesting that young children exhibit greater sensitivity to the relationship between words than that of associated sounds in the semantic system.

The current study aimed at extending these findings, exploring whether words have an advantage over associated sounds in activating conceptual representations in infancy as they have in adulthood. If early in development, infants, like adults, interpret words as abstract, “unmotivated” and arbitrary symbols, and sounds as “motivated” and idiosyncratic cues, the visual object recognition should be modulated by the preceding auditory information. Thus, like in Lupyan and Thompson-Schill [8]study, activation of conceptual representations would be more efficient when target objects are cued by words than by associated sounds. Alternatively, if words and sounds activate conceptual representations similarly, object recognition will not be modulated by its preceding cue. Our first goal was therefore to replicate the study of Lupyan and Thompson-Schill [8] with adults, by using a similar behavioural visual identification task (Experiment 1A). We then conducted an object recognition task with adults (Experiment 1B), measuring eye movements. This eye tracking task was also suitable for infants at 9- (Experiment 2A), 12- (Experiment 2B), and 18 months of age (Experiment 2C). We predicted that adults will react faster to the target image (e.g. cow) when preceded by a spoken word (e.g. “cow”) compared to meaningful associated sounds (e.g. cow mooing). This should also be reflected by faster and longer looking behaviour to the target image (e.g. cow) compared to a distractor (e.g. train). Similarly, we predicted that infants will look faster and longer at the target object when preceded by word compared to associated sound primes, indicating that the advantage of words emerges early during language development.

## Experiment 1A

### Methods

#### Participants

Thirty healthy adults (20 females; age range: 23;2 y to 41;4 y) from the Department of Psychology (*n* = 29) and Computer Science (*n* = 1) volunteered in the experiment. All participants were right-handed. An additional two left-handed participants were excluded from the final sample. Participants were informed about the aim of the study and gave written consent before their participation. The study was approved by the University Research Ethics Committee and conducted in conformity with the declaration of Helsinki.

#### Stimuli

We selected six objects that have basic level nouns and characteristic sounds (car, cow, dog, sheep, telephone, train), suitable for both adults and infants experiments. The auditory stimuli included spoken words and their associated sounds. A native female speaker recorded the words uttered in neutral and adult-directed speech (ADS); and the associated sounds were selected from the internet. Audio files were digitized and edited with Adobe Audition (CS 5.5), at 16-bit resolution and 44 kHz sampling rate and had mean length of 601 ms for words and 883 ms for sounds. The visual stimuli were selected online and included images (see S1 Data) of the six objects, and presented on a 19” CRT monitor.

#### Procedure

The procedure matched closely that of the study by Lupyan and Thompson-Schill (8). Participants sat in front of the monitor and were given a gamepad to respond by button-press. On each trial, participants heard either a word or an associated sound while fixating a central black fixation cross on a grey screen, followed by an image. The inter stimulus interval (ISI) from the offset of the auditory stimulus to the onset of the image was fixed at 1000 ms. The images matched the auditory stimulus 50% of the time, and the order of trials was randomised. Each image remained on the screen for 2 seconds, and participants were instructed to respond as fast as possible by pressing a *match* or *mismatch* button on a gamepad. The side (left and right buttons) of the correct response was counterbalanced across participants. After every response, participants received an auditory feedback, indicating whether their response was correct (a beep) or not (a buzz). As the image disappeared, another trial began. Each of the six objects was preceded by a word or a sound, match and mismatch, and repeated four times, yielding 96 verification trials. The experiment lasted approximately five minutes.

#### Data analysis

Before the analysis, all incorrect responses were removed. As in Lupyan & Thompson-Schill (8), reaction times (RTs) below 200 ms and above 1500 ms were also excluded as well as any trial with no answer (less than 2% of the data, and less than 4% before excluding incorrect trials). The number of trials included was 22 (*SD* = 1.4) for sound-match and 22 (*SD* = 1.8) sound-mismatch, and 22 (*SD* = 1.6) for word-match and 23 (*SD* = 1.1) word-mismatch. RTs and accuracy were analysed with a within-subject *2* (stimulus type: word or sound) x *2* (congruency: match or mismatch) analysis of variance (ANOVA). All data and analysis scripts are available online in https://osf.io/ze429/.

### Results and discussion

**Fig 1:**
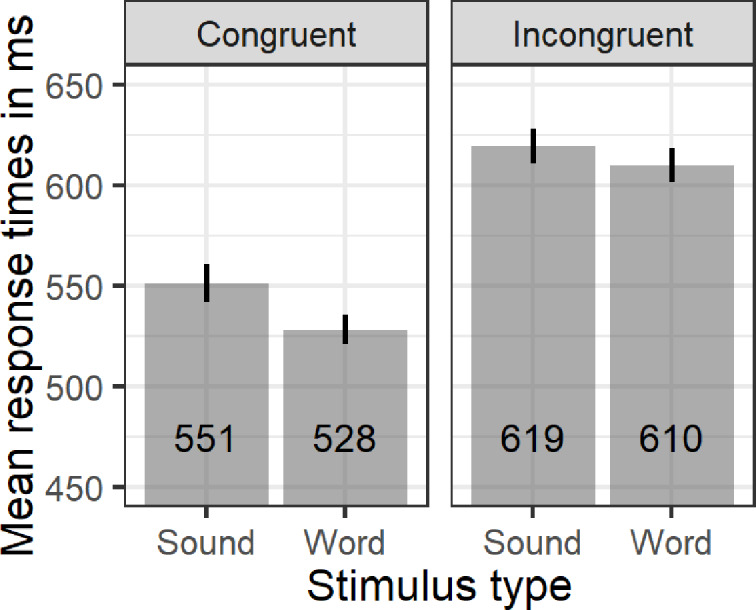
Mean response times (aggregated by participants) as a function of stimulus type and congruency. Error bars without caps represent standard error of the means (SE).

**Fig 2:**
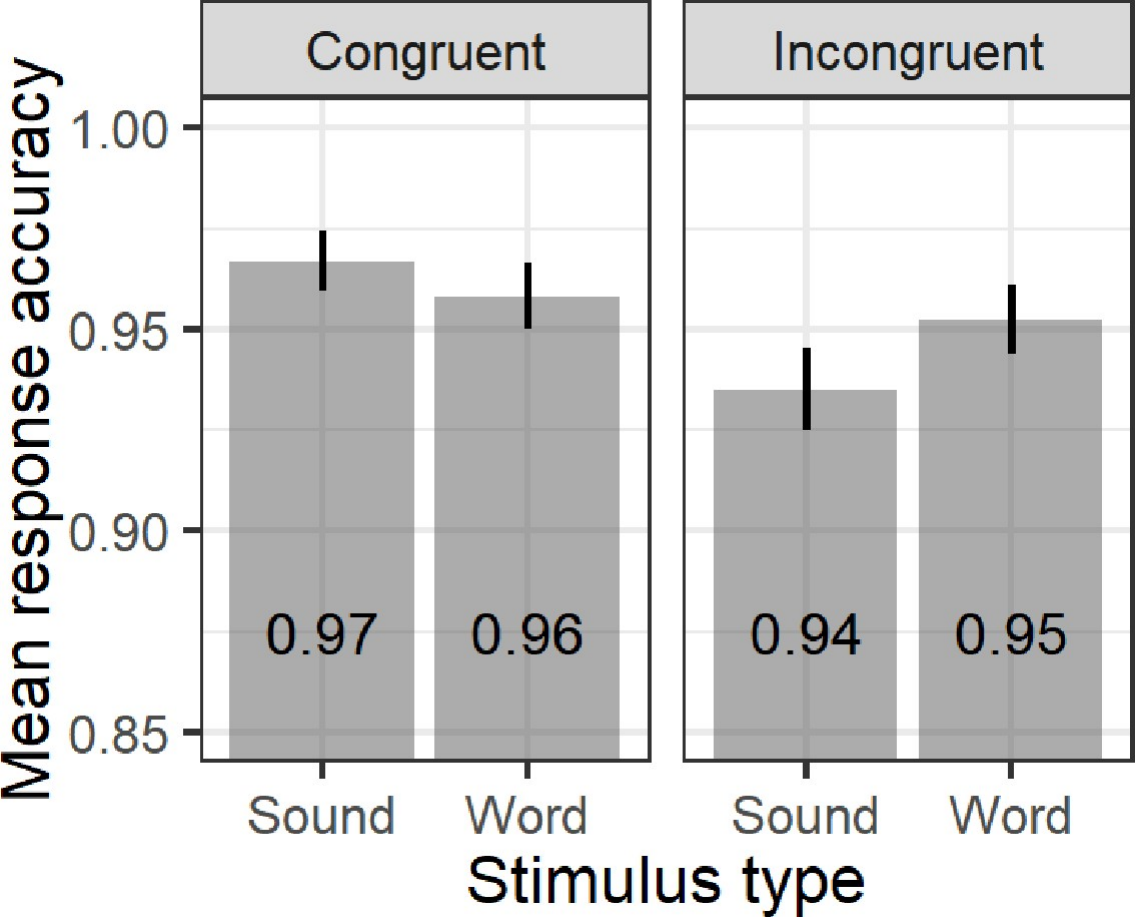
Mean response accuracy (aggregated by participants) as a function of stimulus type and congruency. Error bars without caps represent standard error of the means (SE).

The results showed a marginal main effect of auditory stimulus (*F*(1,29) = 4.11; *p* = 0.051; *η^2^g* = 0.004) and a significant main effect of congruency (*F*(1,29) = 52.35; *p*<0.001; *η^2^g* = 0.08), indicating that for adults, both words and associated sounds activate conceptual representations with greater sensitivity to congruency. Paired sample *t*-test revealed faster responses (*t*(59) = 2.13; *p*<0.05; *Cohen’s d =* 0.27) to words (572 ms; *SD* = 0.11) relative to associated sounds (589 ms; *SD* = 0.13), especially in the congruent trials (*t*(29) = 2.18; *p*<0.05; *Cohen’s d =* 0.39). This advantage of words over sounds is similar to that of Lupyan and Thompson-Schill, (2012, cf. Fig 1). Accuracy analysis revealed significant effect of congruency (*F*(1,29) = 4.93; *p*<0.05; *η^2^g* = 0.04) and an interaction between stimulus type and congruency (*F*(1,29) = 4.63; *p*<0.05; *η^2^g* = 0.02), but no main effect of stimulus type (*F*(1,29)<1). Paired sample *t*-test showed that participants were equally accurate across words and associated sound conditions (97% sound-match; 94% sound-mismatch; 96% word-match; and 95% word-mismatch, cf. Fig 2), but more accurate in the sound-match compared to the sound-mismatch condition (*t*(29) = 3.37; *p*<0.01; *Cohen’s d* = 0.62).

## Experiment 1B

### Participants

Twenty healthy adults (18 female, age range: 24;7 y to 42;7 y) from the Department of Psychology took part in the study, and received £3.50 for their participation.

### Stimuli

The auditory and the visual stimuli were the same used in Experiment 1. The visual stimuli were arranged into 12 images (709 x 425 pixels) of paired objects, one on the left and one on the right side of the screen. Each pair included a target (e.g. dog) and a distractor (e.g. car) from two different semantic categories, presented on a 1920 x 1080 computer screen.

### Procedure

Participants sat at 50–70 cm in front of the computer screen. A Tobii X120 eyetracker (Tobii Pro, Stockholm, Sweden) located beneath the screen recorded their gaze at 60 Hz sampling rate. The eye tracker was first calibrated, using a five-point calibration (shrinking blue and red attention grabber) procedure delivered through Matlab^®^ (v. 2013b). The calibration was controlled with a key press and repeated if necessary. Each trial began with the appearance of a black fixation cross centred on a grey screen for 1000 ms after which an auditory stimulus was played, a word or an associated sound, while the fixation cross remained on the screen. The visual stimulus depicting two objects simultaneously–target and distractor–appeared, and remained on the screen for 2000 ms while the eye tracker recorded participant’s gaze. The inter stimulus interval (ISI) from the offset of the auditory stimulus to the onset of the image was fixed at 1000 ms. After 2000 ms the image disappeared, and another trial began. The side of target and distractor was counterbalanced, resulting in one block of 24 trials. The experimental block was repeated 4 times, yielding 96 trials in total. The order of trials within a block and across participants was randomised. The experiment lasted approximately 9 minutes.

### Data analysis

Two areas of interest that matched size and location of the displayed target and distractor images were defined using Matlab^®^ (v. 2014b), and a summary of participants’ fixations with their duration and coordinates on the display was produced using the same software.

After data pre-processing, we calculated fixation proportions for each of the images on the display in both stimulus type conditions (words vs. sounds) using R software [17]. A value of 1 was given to an object when participants were fixating its region on the display at a given moment, while a value of 0 was given to the other region. If no fixation was detected by the eye tracker, both regions were given a 0 value. We defined fixation proportion as the percentage of looks to an object on each trial and across time. This measure was then aggregated, first by participant and stimulus type, and then into 100 ms time windows. The first aggregation allows us to calculate confidence intervals, which were corrected for within-subject designs and for number of multiple comparisons. The second aggregation helps to lessen auto-correlation between fixation proportions over time.

To evaluate the effects of words and sounds on participants’ looks to the pictures on the display, we used a complementary approach based on confidence intervals and quantifiable effect size of proportion of fixation over time [18, 19, 20], plus a quasi-logistic growth curve analysis (GCA) approach [21, 22, 23] on empirical logit transformation of the proportion of fixations [24, 25]. These two analyses allow complementary inferences by tackling different aspects of eye tracking data in the VWP. Following Baayen [26], we considered all t-values > |2| as significant effects (e.g. *p*<0.05).

### Results and discussion

**Fig 3:**
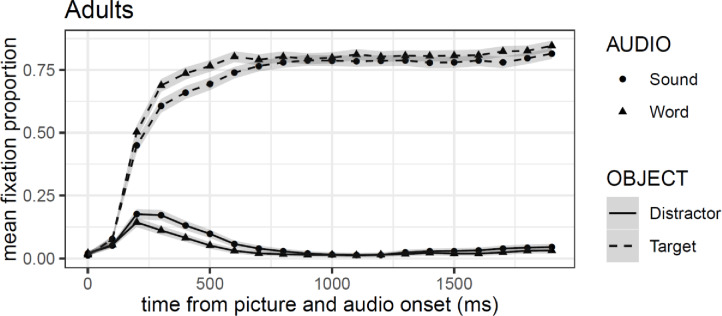
Mean fixation proportion (aggregated by participants) as a function of object in the visual context and type of auditory stimulus in Experiment 1B. Shaded areas around lines represented 95% confidence intervals adjusted for within-subject designs and multiple time windows.

Fig 3 shows mean proportion of fixation by object and stimulus type. Shaded areas around the lines represent the within-subject adjusted 95% confidence intervals. Points mark 100 ms time bins from the onset of auditory stimuli windows and distinguish between types of stimulus (i.e. words vs. sounds). The results show greater preference for the target objects, both when hearing the label of the object (word) and its associated sound. This preference for the target over the distractor was also independent of the nature of the item, animals or objects (see S1 Data). Fig 3 shows that this preference is slightly stronger for the words compared to the associated sounds. After 200 ms from stimuli onset, a larger fixation proportion on the target object is observed when participants heard the label of the object. This advantage is evident for about 400 ms, disappearing around 700 ms after stimuli onset.

**Fig 4:**
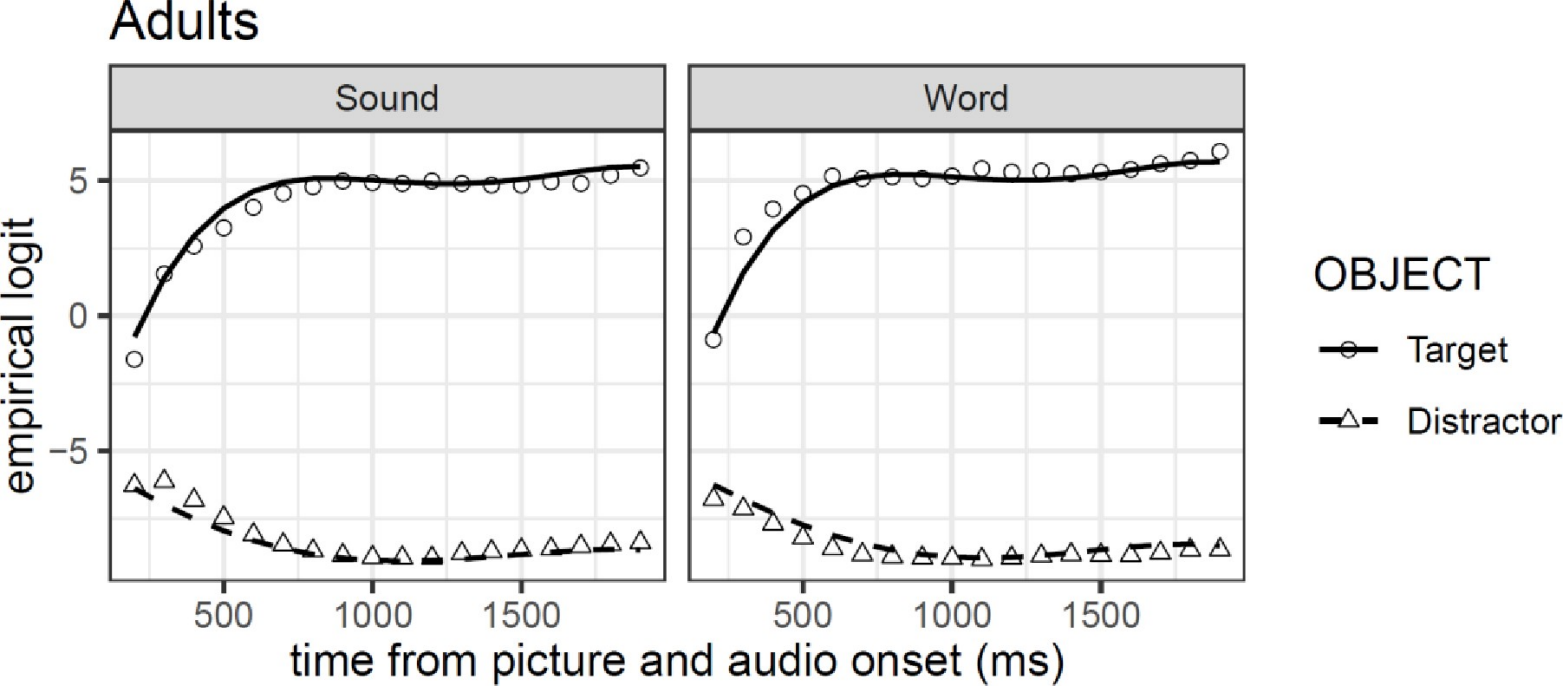
GCA model fit (lines) of empirical logit (points) as a function of object in the visual context and type of auditory stimulus in Experiment 1B.

Model comparison for Experiment 1B resulted in the selection of a model that included all four orthogonal polynomial terms (all *χ²*-values > 142.56, *df* = 11, all *p*-values > .001). The results of the GCA model are presented in Table 1. All polynomial terms show reliable main effects and interaction with the difference between objects (target vs. competitor), except for the quadratic polynomial, which exhibit only the interaction but no main effect in the model. Model comparison, nonetheless, shows that a model with all four terms produce a better fit of the data relative to one without the quadratic term (*χ²* = 2633.5, *df* = 11, all *p*-values > .001). As expected, based on the confidence intervals analysis, the results of the GCA model showed a reliable main effect of object (*β* = -6.41, *se* = 0.28, *t* = -22.71), however, the word preference is not captured in the model (*t* < |2|). Fig 4 shows that the shape of the fixations towards the targets assumes a quartic form with an initial quadratic form, in contrast to the gaze pattern to distractors, which takes a more pronounced cubic and linear shape.

**Table 1:** Main and interaction effect in the quasi-logistic GCA mixed model analysis in Experiment 1B.

	*Estimate*	*se*	*t*	
(Intercept)	-1.96	0.38	-5.10	*
Linear	1.36	0.59	2.29	*
Quadratic	-0.60	0.49	-1.24	
Cubic	1.10	0.20	5.46	*
Quartic	-0.68	0.12	-5.44	*
Object	-6.41	0.28	-22.71	*
Condition	-0.08	0.12	-0.69	
Linear * Object	-3.54	0.41	-8.63	*
Linear * Condition	0.01	0.23	0.03	
Quadratic * Object	2.83	0.47	6.03	*
Quadratic * Condition	-0.03	0.14	-0.22	
Cubic * Object	-1.76	0.19	-9.40	*
Cubic * Condition	-0.02	0.10	-0.18	
Quartic * Object	0.50	0.12	3.99	*
Quartic * Condition	0.06	0.08	0.76	

* = *p*<0.05

These results strengthen and support our replication in Exp. 1A by showing that adults looked faster at the target object in the word compared to the sound condition. This preference for words was also reflected by longer early looking time to the target in response to words. The analysis of mean looking times revealed that longer looking to the target was more prominent in the words compared to the associated sounds condition (see S1 Data).

Both experiments further confirm the theory that conceptual representations are activated more effectively through verbal (words) than nonverbal (associated sounds) means, suggesting that words exert stronger effect on the activation of visual components of the related conceptual representations.

The question of whether this phenomenon emerges early in infancy is studied in the following set of experiments. Previous developmental studies have shown that words, compared to non-linguistic sounds, enhance object categorization (3, for a review) in infants. And, under specific circumstances (e.g. mother’s voice or presenting two objects from different categories), 9-month-old infants have the capacity to understand the meaning of some common words [27], and detect the match or mismatch between the auditory label and visual object [28]. By 18 months, infants are more sensitive to the relationship between words (e.g. dog–“cat”) than that of associated sounds (e.g. dog–meowing) [16]. We therefore hypothesized that at 9 months, words will have an advantage over associated sounds in activating conceptual representations. We expected infants to look faster and longer at the target relative to the distractor object, in particular, when preceded by words compared to associated sounds.

## Experiment 2

### Methods

#### Participants

Thirty-two healthy 9-month-old infants (15 girls; age range: 8m3d to 9m23d) took part in Exp. 2A. In Exp. 2B, there were thirty-two 12-month-olds (17 girls; age range: 11m2d to 12m23d), and in Exp. 2C twenty-three 18-month-old (12 girls; age range: 17m14 to 18m8d) infants. Participants were recruited from a database of parents from the local area who expressed an interest in taking part in developmental research studies. Parents were informed about the aim of the study and gave written consent before participation. An additional forty infants took part in the study but were not included in the final sample due to an insufficient amount of trials per condition (word or sound; *n* = 35), no familiarization phase (*n* = 1), participating twice (at 9- and 12 months; *n* = 1), low birth weight (<2500 kg; *n* = 2) or premature (<37 weeks of gestation; *n* = 1). All infants received a book for their participation and parents were reimbursed £10 for travel expenses. The study was approved by the University Research Ethics Committee and conducted in conformity with the declaration of Helsinki.

#### Stimuli

The auditory stimuli were the basic level spoken words and their associated sounds as in Experiment 1. A different native female speaker recorded the words uttered in infant-directed speech (IDS). Audio files were digitized and edited with Adobe Audition (CS 5.5), at 16-bit resolution and 44 kHz sampling rate and had mean length of 819 ms for words and 883 ms for sounds. The visual stimuli were the same 24 images from Experiment 1B.

#### Procedure and data analysis

We adapted the procedure from Experiment 1B to infants, by adding a familiarization phase (using slide presentation (Microsoft Office 2016) on an iPad mini (7,9”) tablet); and, by increasing the time of the fixation cross on the screen to 3000 ms. During this time, caregivers were encouraged to maintain their infant’s attention and interest in the task by saying for instance, “*Oh look*!” or “*Look …*.*”*. Infants sat on their caregiver’s laps, and caregivers were asked to sit at a 90° angle from their infant to ensure the eye tracker recorded the infants’ eye movements only, and to facilitate the interaction between trials. Caregivers were also instructed to avoid verbal communication when the auditory and visual stimuli were displayed, pointing to the screen or naming the objects. The visual stimulus remained on the screen for 4.5 seconds while the eye tracker recorded infants’ gaze. After 4.5 seconds, the image disappeared, and another trial began. Infants were presented with one block of 24 trials in total. A break was taken when needed, and the experiment lasted approximately 5 minutes. The data analysis was identical to that of Experiment 1B, and was applied to each of the age group separately.

### Results and discussion

#### Experiment 2A: 9-month-olds

**Fig 5:**
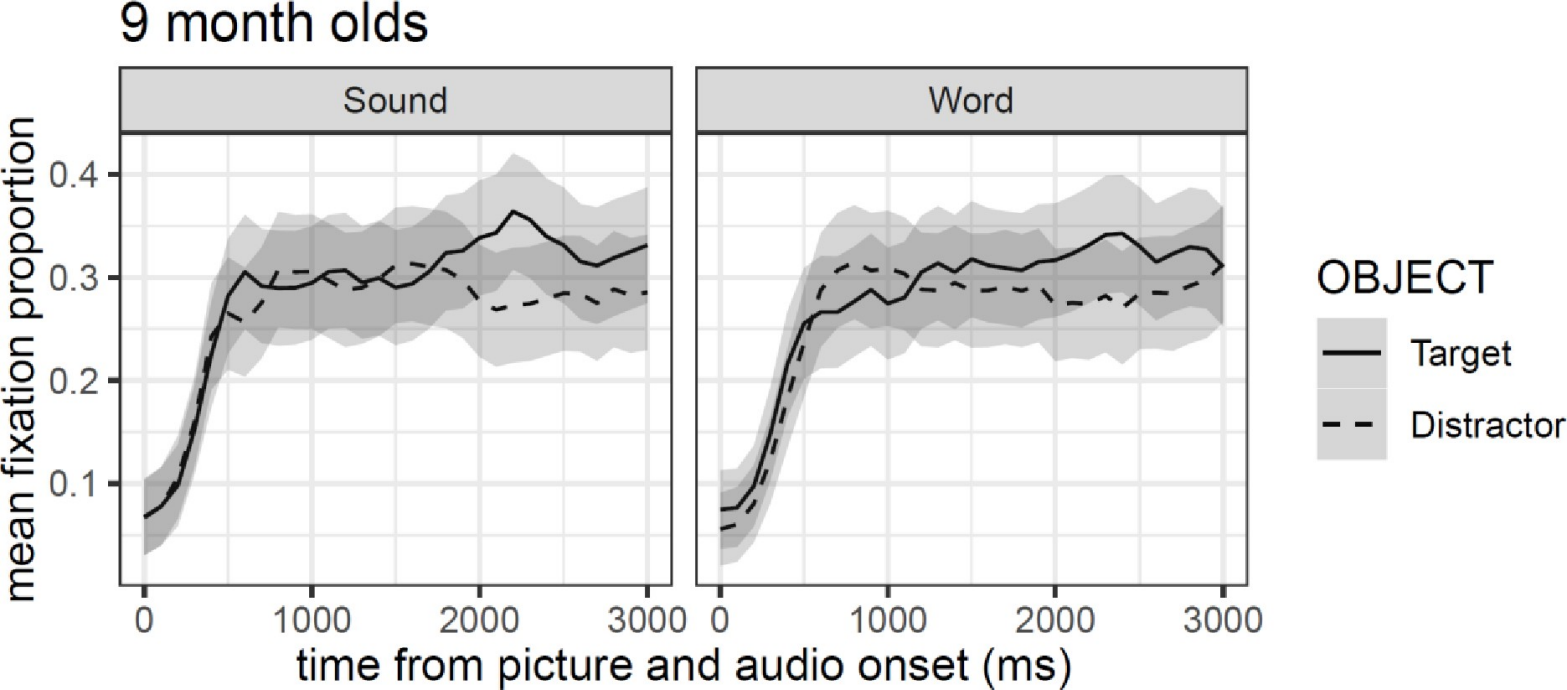
Mean fixation proportion (aggregated by participants) as a function of object in the visual context and type of auditory stimulus. Shaded areas around lines represented 95% confidence intervals adjusted for within-subject designs and multiple time windows.

Fig 5 reveals that target objects were preferred relative to the distractors, particularly between 2000 ms and 2500 ms. However, the confidence intervals suggest that this effect is too small to be considered significant. Similarly, the gaze pattern to the target does not appear to differ between stimulus types. We now turn to the GCA approach to corroborate these results.

An adult saccadic eye movement is generally assumed to take about 200 ms [29, 30, 31]. Arguably, however, children take longer than adults to program and initiate saccades [32]. Thus, GCA regressions consider time windows from 300 ms to 3000 ms after the onset of the stimuli. The results show significant main effects of all polynomial terms, reflecting that the overall changes over time in the fixation of proportion can be reliably depicted by linear, quadratic, cubic and quartic components (all *t*-values > |2|).

**Fig 6:**
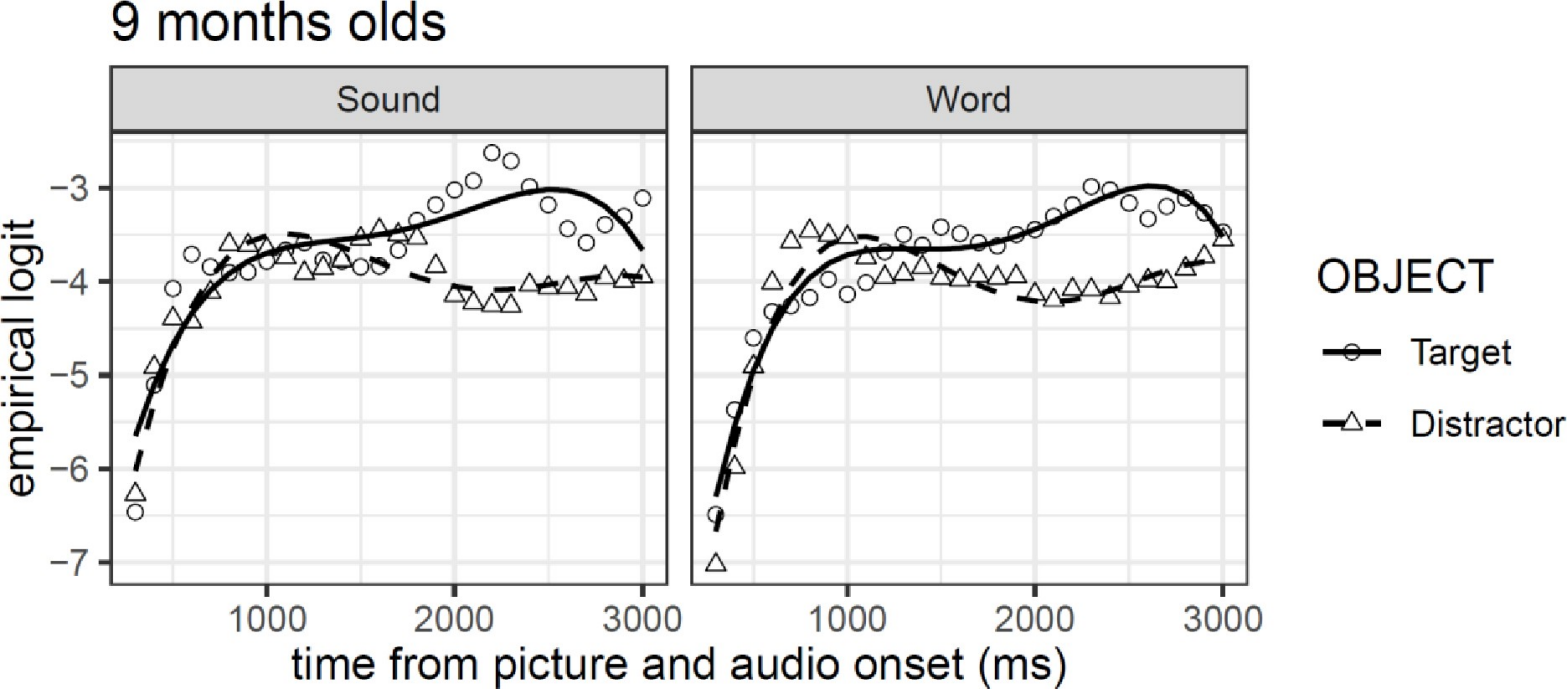
GCA model fit (lines) of empirical logit (points) as a function of object in the visual context and type of auditory stimulus.

More important, the model shows no reliable differences between conditions or objects (both *t*-values < |2|), corroborating the conclusions inferred in the first analysis approach. However, the interaction effect between third-order polynomial predictor of changes over time and object, we found a reliable effect (*β* = 0.73, *se* = 0.32, *t* = 2.25), suggesting subtler overall differences in the time course of looks for target and distractors objects (see Table 2). Fig 6 shows GCA model fits on empirical log data time-locked to 300 ms after stimuli onset. The graph is divided into panels per condition where lines represent GCA model fits (solid for the target and dashed for the distractors), and points represents the empirical logit data per condition (circles for the target and triangles for the distractors). In sum, the results of both analyses suggest a subtle preference for the target object in both the stimulus type experimental conditions, despite displaying no differences between conditions.

**Table 2:** Main and interaction effect in the quasi-logistic GCA mixed model analysis.

	*Estimate*	*se*	*t*	
(Intercept)	-3.89	0.63	-6.12	*
Linear	2.04	0.79	2.57	*
Quadratic	-1.74	0.50	-3.46	*
Cubic	1.40	0.38	3.67	*
Quartic	-1.02	0.30	-3.38	*
Object	-0.19	0.19	-1.01	
Condition	0.05	0.14	0.34	
Linear * Object	-0.86	0.66	-1.31	
Linear * Condition	-0.29	0.42	-0.71	
Quadratic * Object	0.00	0.45	0.01	
Quadratic * Condition	0.03	0.28	0.12	
Cubic * Object	0.73	0.32	2.25	*
Cubic * Condition	-0.26	0.27	-0.95	
Quartic * Object	0.07	0.27	0.25	
Quartic * Condition	0.17	0.22	0.76	

* = *p*<0.05

These findings are not in line with our prediction that word advantage emerges at 9 months when infants show semantic understanding of common words. Nine-month-old infants recognized the visual target object, however, looking time and fixations were similar across conditions. Consequently, we hypothesized that words will become more effective at activating conceptual representations at 12 months, when their mental representation of words as abstract referential symbols might be more consolidated.

### Experiment 2B: 12-month-olds

**Fig 7:**
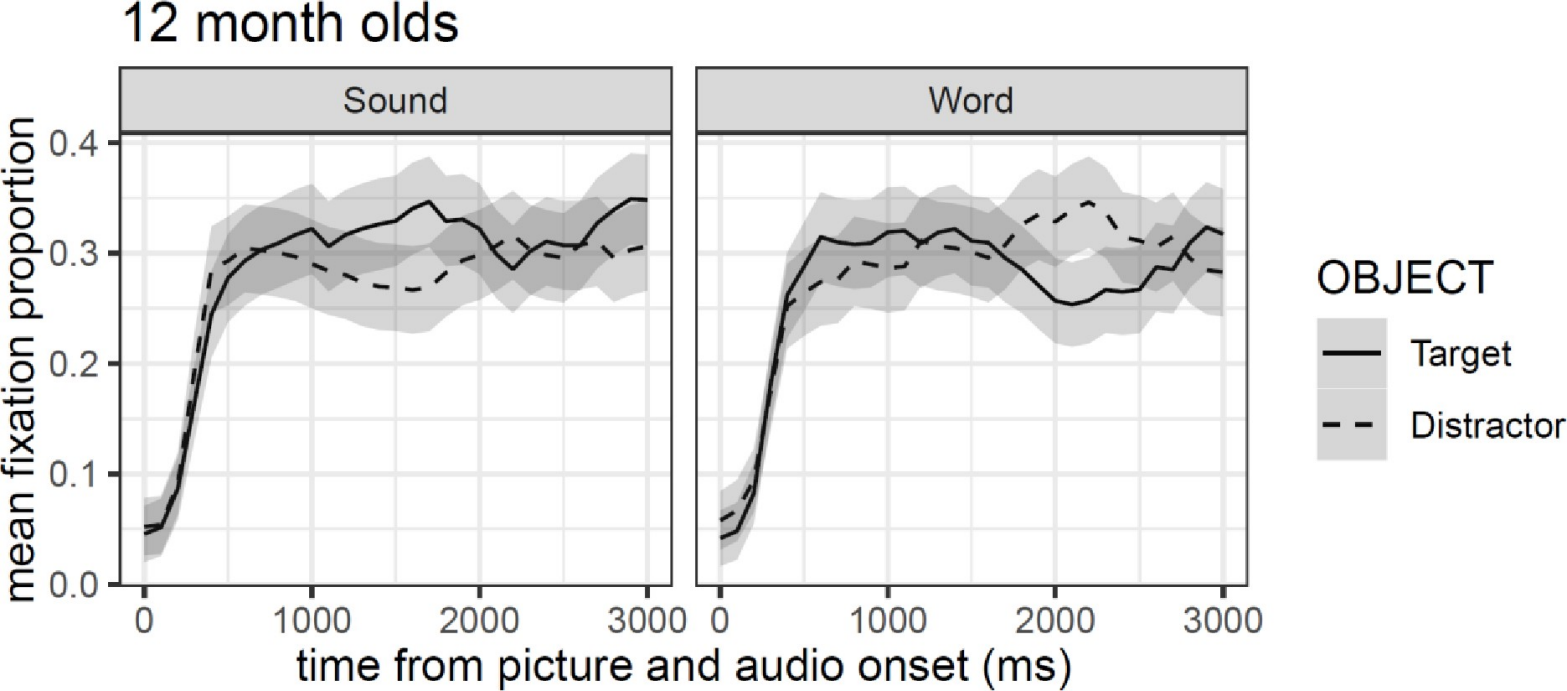
Mean fixation proportion (aggregated by participants) as a function of object in the visual context and type of auditory stimulus. Shaded areas around lines represented 95% confidence intervals adjusted for within-subject designs and multiple time windows.

Fig 7 shows a distinctive pattern for the sound and the word experimental conditions. Target objects show a small and short-lived preferences in the sound condition with a peak around 1750 ms after stimuli onset, while a similar pattern is observed for the distractor in the word condition but with a later peak (around 2150 ms after stimuli onset). Confidence intervals, however, suggest that these effects are too small to be considered significant.

**Fig 8:**
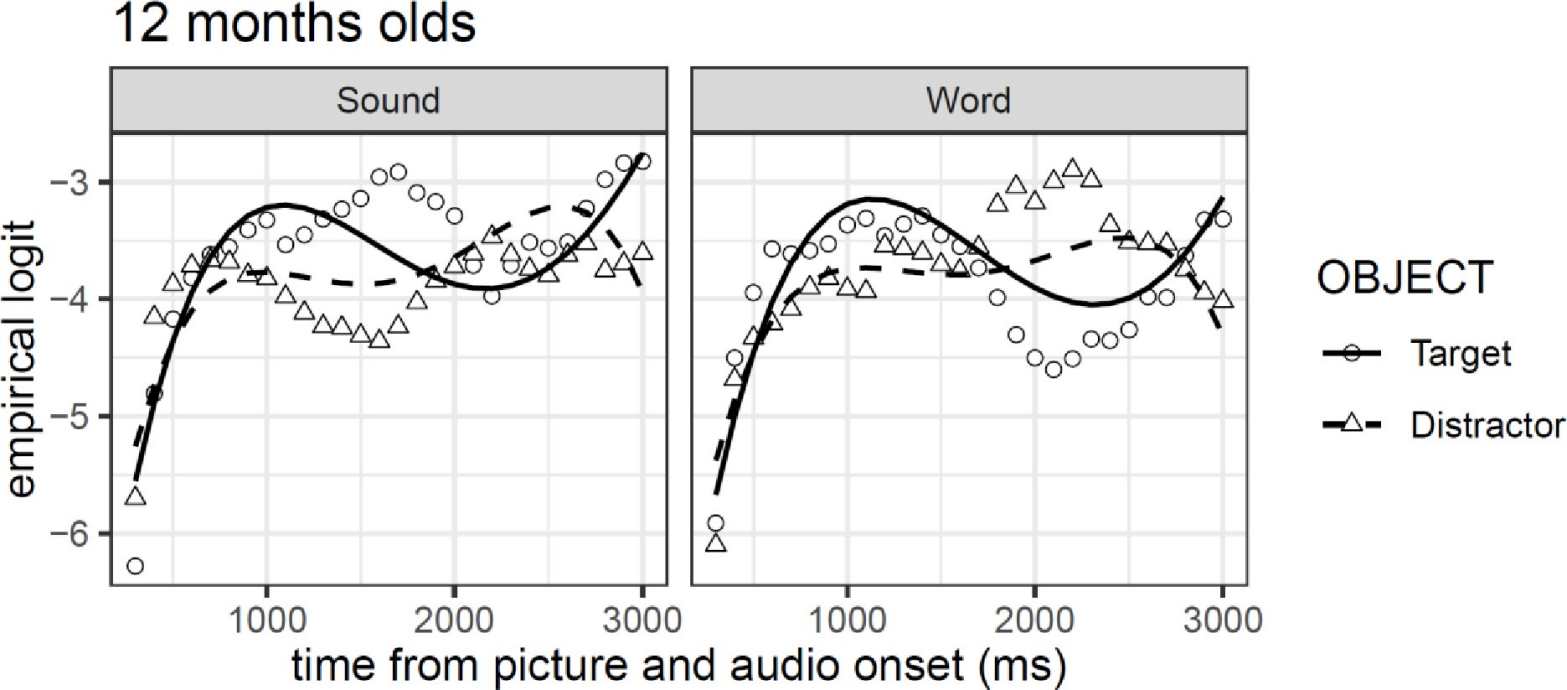
GCA model fit (lines) of empirical logit (points) as a function of object in the visual context and type of auditory stimulus.

Model comparisons showed that the inclusion of each polynomial term increased the fit of the model (all *χ²*-values > 51.86, *df* = 11, all *p*-values > .001). Critically, and as for the 9-month-olds, we observed no reliable overall differences between objects or conditions, and an interaction between the cubic polynomial and object (see Table 3). However, the pattern observed is different to that in 9 months old children. As it can be observed in Fig 8, the model fit for the target in both conditions takes the form of a cubic curve, while that for the distractor can be better described as a quartic curve. Consequently, the combination of the two analyses approach suggests that as in Exp. 2A, there are no differences between the experimental conditions, and that there might be subtle differences between the time course visual attention pattern for target objects and distractors.

**Table 3:** Main and interaction effect in the quasi-logistic GCA mixed model analysis in Experiment 2B.

	*Estimate*	*se*	*t*	
(Intercept)	-3.77	0.67	-5.63	*
Linear	1.30	0.75	1.74	
Quadratic	-1.13	0.52	-2.17	*
Cubic	1.38	0.41	3.39	*
Quartic	-0.75	0.35	-2.13	*
Object	-0.06	0.11	-0.56	
Condition	0.04	0.13	0.33	
Linear * Object	0.20	0.52	0.39	
Linear * Condition	0.25	0.46	0.55	
Quadratic * Object	-0.05	0.38	-0.13	
Quadratic * Condition	0.29	0.40	0.73	
Cubic * Object	-1.08	0.37	-2.90	*
Cubic * Condition	-0.04	0.24	-0.17	
Quartic * Object	-0.37	0.23	-1.58	
Quartic * Condition	-0.08	0.32	-0.26	

* = *p*<0.05

Unexpectedly, we obtained similar results to Exp. 2A. Twelve-month-old infants did not show a preference for words over associated sounds during object recognition. However, for each age group, 9 and 12 months, the analysis per item and mean proportion of fixtaions provided a slightly clearer pattern, and revealed that infants looked longer at the target compared to the distractor only when items were animals as opposed to objects (see S1 Data). This preference was independent of the conditions, words or associated sounds, and could be explained either by familiarity or by animacy.

Earlier studies have shown that the second year is marked by an accelerated rate of word learning and understanding, yielding a more efficient recognition [33], and greater sensitivity to the relationships between words than that of associated sounds []16]. Thus, we hypothesized that at 18 months, infants will exhibit greater sensitivity to words, and visual object recognition will be more effective when cued by words than associated sounds.

### Experiment 2C: 18-month-olds

**Fig 9:**
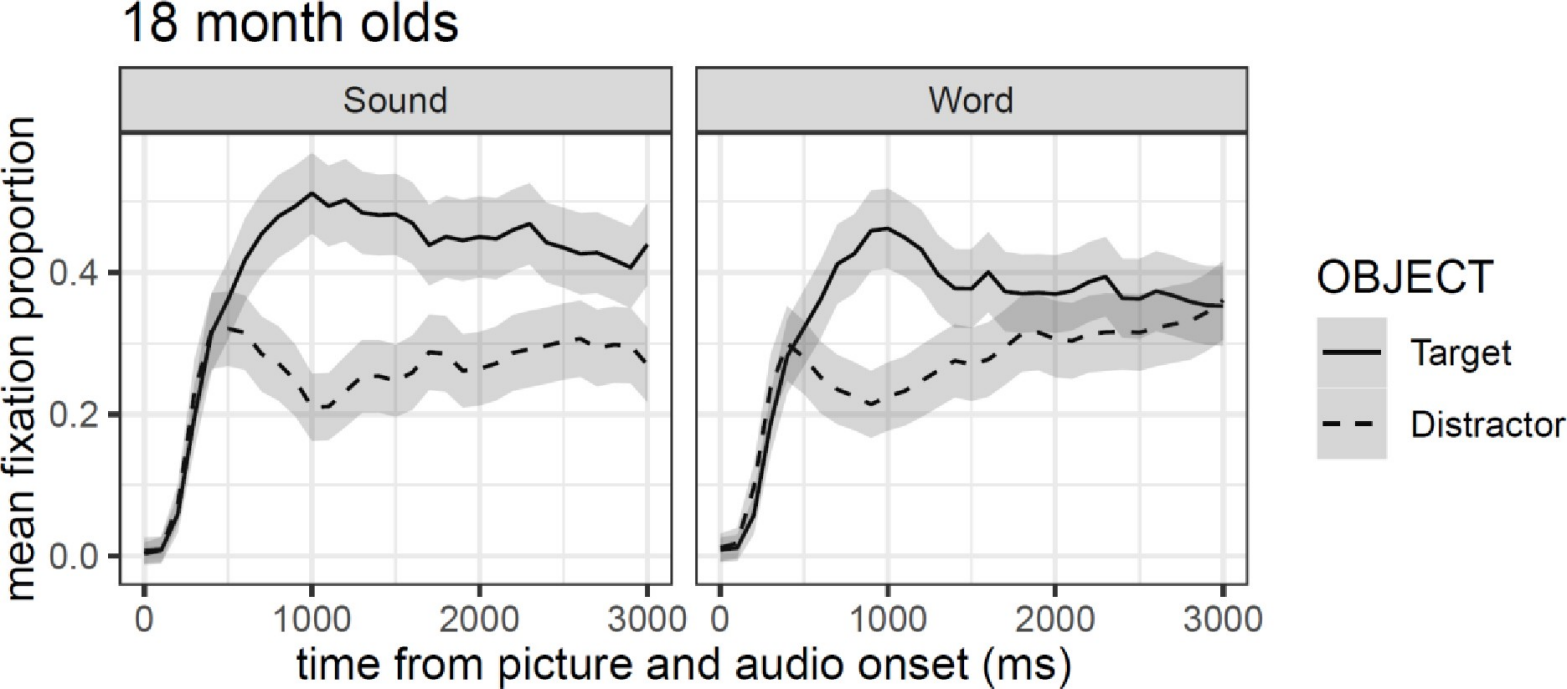
Mean fixation proportion (aggregated by participants) as a function of object in the visual context and type of auditory stimulus. Shaded areas around lines represented 95% confidence intervals adjusted for within-subject designs and multiple time windows.

In contrast to Exp. 2A and Exp. 2B, the pattern of fixation proportion in Fig 9 shows a clear preference for the target object (compared to the distractor) in both conditions. This preference starts around 600 ms after stimuli onset and it is maintained beyond 2500 ms after word onset in the sound condition, but only until 1500 ms after word onset in the word condition. Nevertheless, the confidence intervals suggest that while a larger difference between target and distractors is evident in the sound condition relative to the word condition, there is no clear differences between the two experimental conditions.

**Fig 10:**
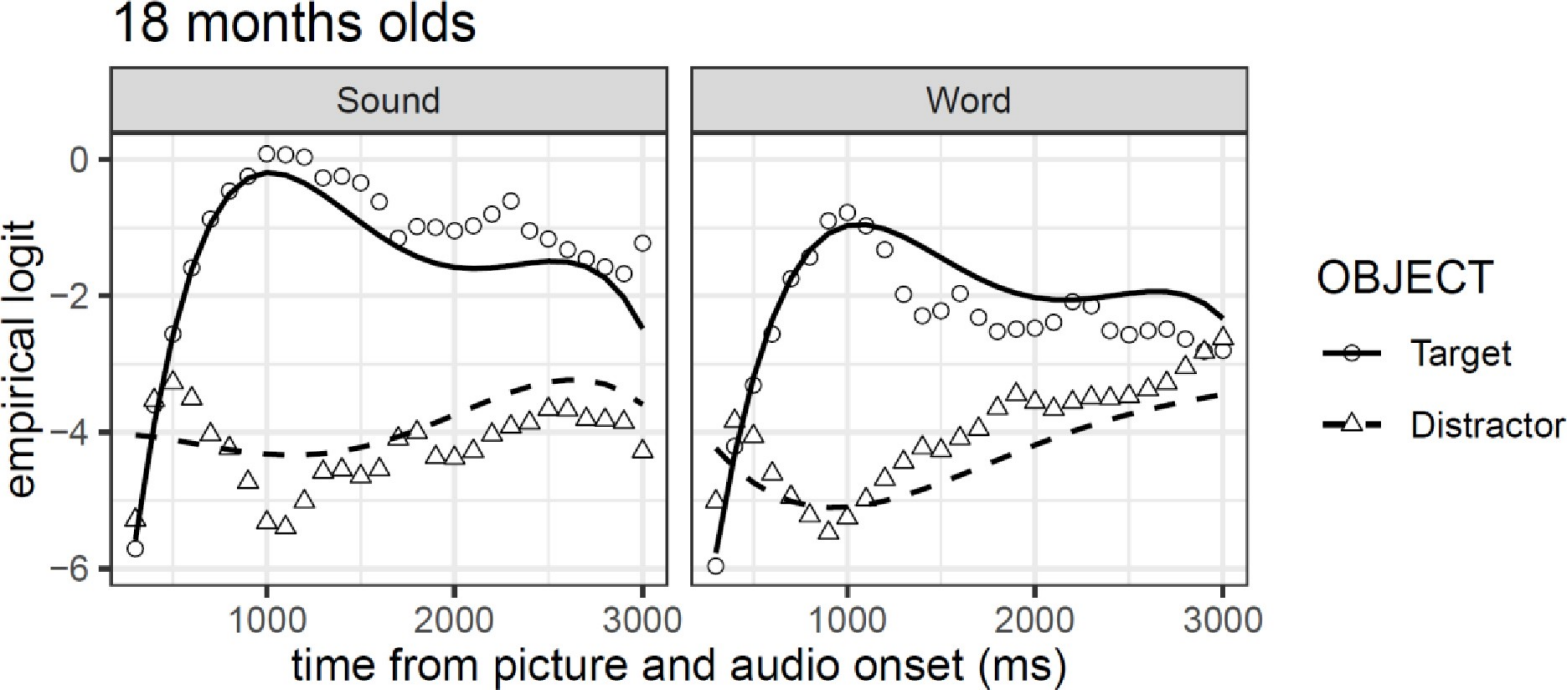
GCA model fit (lines) of empirical logit (points) as a function of object in the visual context and type of auditory stimulus in Experiment 2C.

Model comparison resulted in the selection of a model that included all four orthogonal polynomial terms (all *χ²*-values > 63.84, *df* = 11, all *p*-values > .001). Importantly, and in contrast to Exp. 2A and Exp. 2B, the results of the GCA model showed a reliable main effect of object (*β* = -1.19, *se* = 0.30, *t* = -3.96), but no reliable main effect of condition (*t* < |2|). This is coherent with what can be inferred based on the confidence intervals approach (see Fig 10). Additionally, the GCA model shows three significant interaction effects between object and the polynomials terms quadratic, cubic, and quartic (see Table 4). Fig 10 shows that the shape of the fixations on the targets over time takes a quartic form with an initial strong quadratic shape. In contrast, the fixation to distractors assume a much more pronounced cubic shape relative to the target objects.

**Table 4:** Main and interaction effect in the quasi-logistic GCA mixed model analysis.

	*Estimate*	*se*	*T*	
(Intercept)	-2.94	0.56	-5.26	*
Linear	1.52	0.98	1.55	
Quadratic	-1.41	0.73	-1.93	
Cubic	1.22	0.55	2.23	*
Quartic	-1.15	0.44	-2.60	*
Object	-1.19	0.30	-3.96	*
Condition	0.25	0.21	1.20	
Linear * Object	0.61	0.71	0.86	
Linear * Condition	-0.37	0.72	-0.52	
Quadratic * Object	2.31	0.61	3.80	*
Quadratic * Condition	-0.27	0.39	-0.69	
Cubic * Object	-2.12	0.50	-4.21	*
Cubic * Condition	0.08	0.32	0.25	
Quartic * Object	1.04	0.39	2.68	*
Quartic * Condition	-0.34	0.25	-1.36	

* = *p*<0.05

These results show that 18-month-olds were equally fast at recognizing the target object in both the word and sound conditions, and independently of the nature of items (animals or objects; see S1 Data). The difference between both conditions was not significant, but as reflected in the GCA model, infants displayed longer looking time in the associated sound compared to the word condition.

## General discussion

In this study, we aimed to determine whether during language development, words are more effective than associated sounds at activating conceptual representations. We conducted one behavioural visual identification and one eye tracker object recognition experiment with adults to replicate Lupyan and colleagues’ [8]findings. We then adapted the object recognition task so that it was suitable for infants. Our successful replication revealed that adults identified and recognized faster the target object when preceded by its label compared to its associated sound, supporting further the theory that although both words and associated sounds activate conceptual representations, words have the advantage of being more efficient in activating the visual representation of an object. In Lupyan and Thompson-Schill’s study [8], the word advantage was also evident in the accuracy measure, whereas in our study, participants were equally accurate across words and associated sounds conditions. The eye movement measures, however, strengthened the RTs findings and yielded a similar word advantage.

It is possible that upon hearing the word “*dog*” for instance, all dog features including their generic visual appearance are activated, accelerating the reaction times, while hearing dog barking might require increased verification time to create the direct link between the source of the sound and the image. This is unlikely, however, as even with a longer average sound duration (relative to word duration) providing participants with additional processing time, RTs and looking times were faster in the word compared to the associated sound condition. Thus, unlike associated sounds, words enhance the abstraction of conceptual categories leading to faster activation of the category representations: words are “special” because they enable activation of conceptual representations in a more categorical way [3, 8].

However, this phenomenon did not emerge in our experiments with infants at 9-, 12-, or 18-months. Nine- and 12-month-olds did not display any differences between words and associated sounds conditions. Moreover, the distinction between the target and distractor object in either condition was not reliable. Consequently, it can be assumed that at these ages, infants process both auditory stimuli differently, but our empirical paradigm was not sensitive enough to detect these nuances. Unexpectedly, although 18-month-old infants were equally fast at recognizing the target object in both conditions, we observed a shift towards larger fixations and longer looking time at the target when preceded by the associated sound compared to the word. Our findings suggest that infants allocated greater attention to the target image in the associated sound compared to the word condition. This result must be taken with caution and needs to be considered carefully. Though it matches Hendrickson et al. [16]suggestion that associated sounds require longer time to process the semantic match between the visual object and the generated sound, it contradicts the results of Cummings and colleagues [15], whereby object recognition was similar in the words and associated sounds condition. Toom and Kukona’s [11]VWP study with adults, found greater looking times and semantic activation of the competitors in the associated sounds relative to the words conditions. Just like in our study, however, this source of differences requires further replication and investigation.

Alternatively, developmental studies commonly claim that words have a special status and are preferred over other non-linguistic sounds because of their *referential* nature [3]. In this account, words enhance categorization and learning because unlike other sounds, words *refer* to object kinds. Therefore, it could be that during infancy, words activate conceptual representations more efficiently than associated sounds, but another methodology would be more sensitive to these differences. We also want to note that, although the ISI was kept constant (1000 ms), for both adults and infants, the average duration of words was shorter compared to sounds, and participants had more time to process sounds over words. As Exp. 1B indicates, this had no effect on adult results but might have had an effect on infant results.

Another possibility is that words become more effective at activating conceptual representations, but their advantage over associated sounds emerges at later stages of language development. The only two studies to date that investigated the processing of words and associated sounds tackled the question differentially. Cummings et al. [15]studied the speed of word-object recognition and its correlation with chronological age and infants’ productive skills, while Hendrickson et al. [16]investigated the organization of words and associated sounds in the semantic memory. Here, we were interested in studying whether visual object recognition is modulated by the preceding auditory cue to determine whether words have a ‘special’ status compared to associated sounds.

Our study demonstrates that by 18 months, infants process words and associated sounds differently, possibly allocating more attention to target objects when cues by associated sounds relative to words. The question of whether and when infants reach the pattern of results we observed in adults remains open: a different experimental methodology or different ages might yield the initially expected results.

## Supporting information

S1 Data (DOCX)
