# Supplementary material for: Infants’ conceptual representations of meaningful verbal and nonverbal sounds
Source: PLoS One. 2020 Jun 8;15(6):e0233968. doi: 10.1371/journal.pone.0233968 (PMC7279894; doi:10.1371/journal.pone.0233968)

**Infants’ conceptual representations by meaningful verbal and nonverbal sounds**

Louah Sirri, Ernesto Guerra, Szilvia Linnert, Eleanor S. Smith, Vincent Reid, & Eugenio Parise

**Supplementary Materials**

Content

1. Analysis per item

2. Analysis per semantic category and corresponding Growth Curve Analysis

3. Total looking times

**1. Analysis per item**

The results showed that adults (Fig. A1) show a clear preference for the target over the distractor object, independently of the nature of the item (animals or objects). Nine- (Fig. A2) and 12-month-old (Fig. A3) infants, however, preferred images depicting animals (cow, dog, sheep) relative to the objects (car, telephone, train), independently of the auditory stimulus condition (words *vs* associated sounds). Eighteen-month-olds, on the other hand, exhibited an overall preference for the target object, yet this preference is clearer for animal relative to objects (Fig. A4). These findings are interesting, and could be either explained by familiarity or animacy.

- 1. **Experiment 1A: Adults**


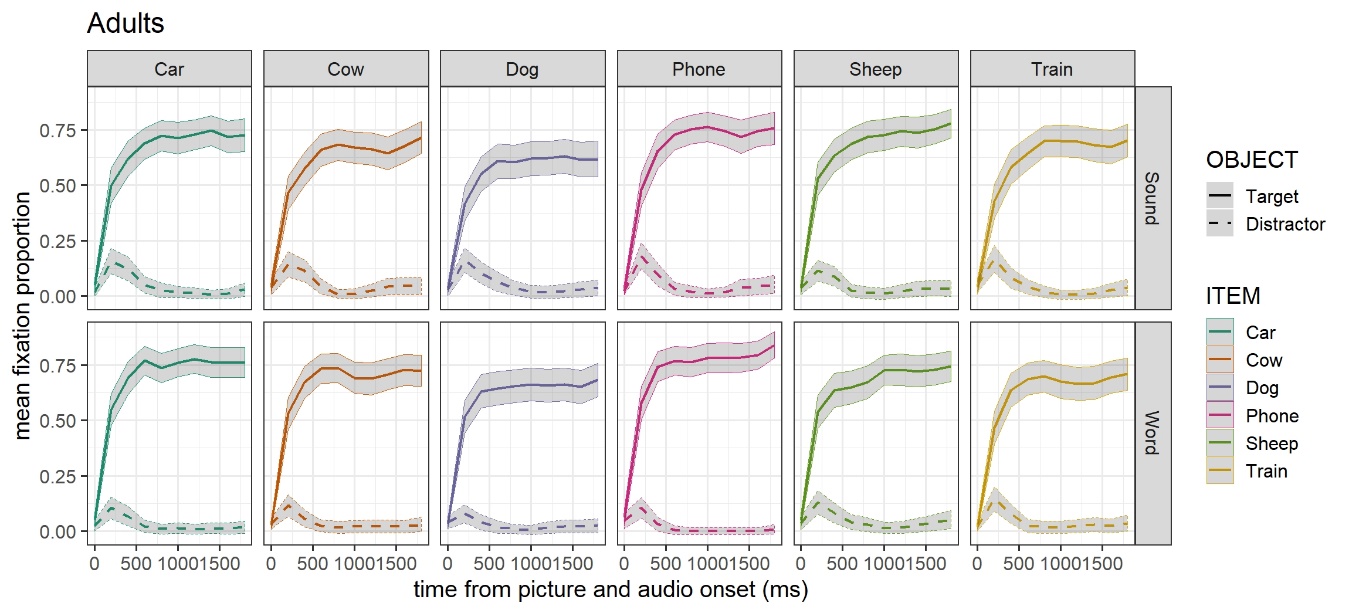


**Figure A1.** Mean fixation proportion (aggregated by participants) as a function of object in the visual context, type of auditory stimulus and individual items in Experiment 1B. Shaded areas around lines represented 95% confidence intervals adjusted for within-subject designs and multiple time windows.

**1.2. Experiment 2A: 9-month-old infants**


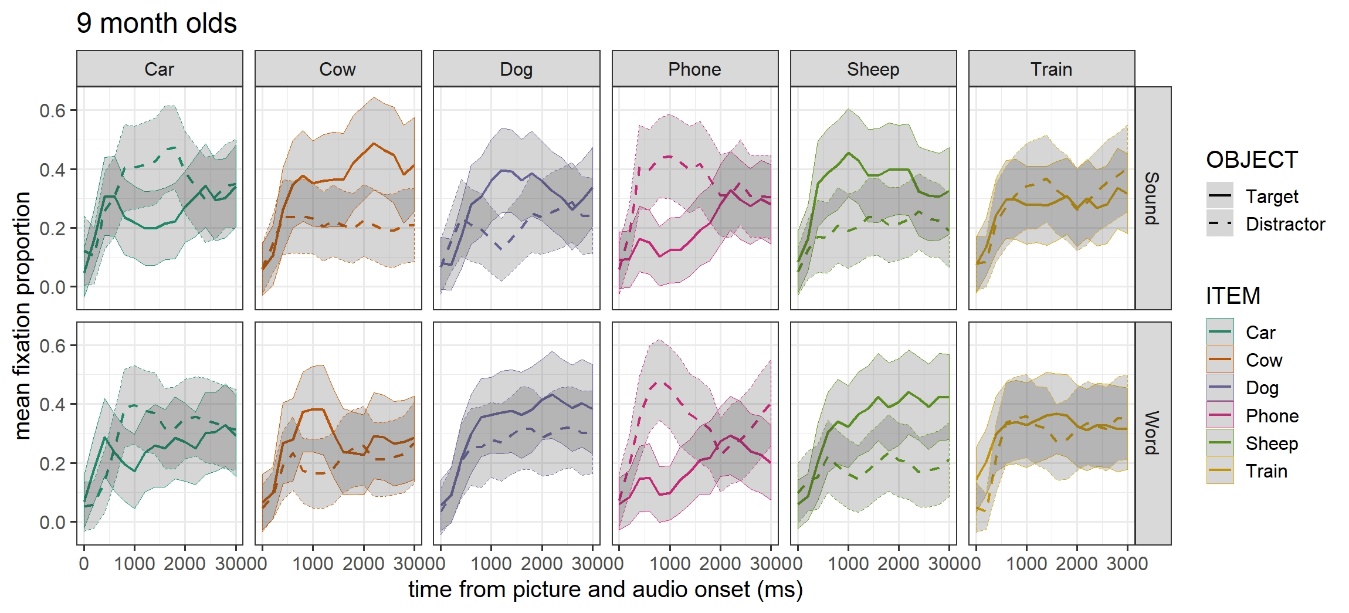


**Figure A2.** Mean fixation proportion (aggregated by participants) as a function of object in the visual context, type of auditory stimulus and individual items in Experiment 2A. Shaded areas around lines represented 95% confidence intervals adjusted for within-subject designs and multiple time windows.

**1.2. Experiment 2B: 12-month-old infants**


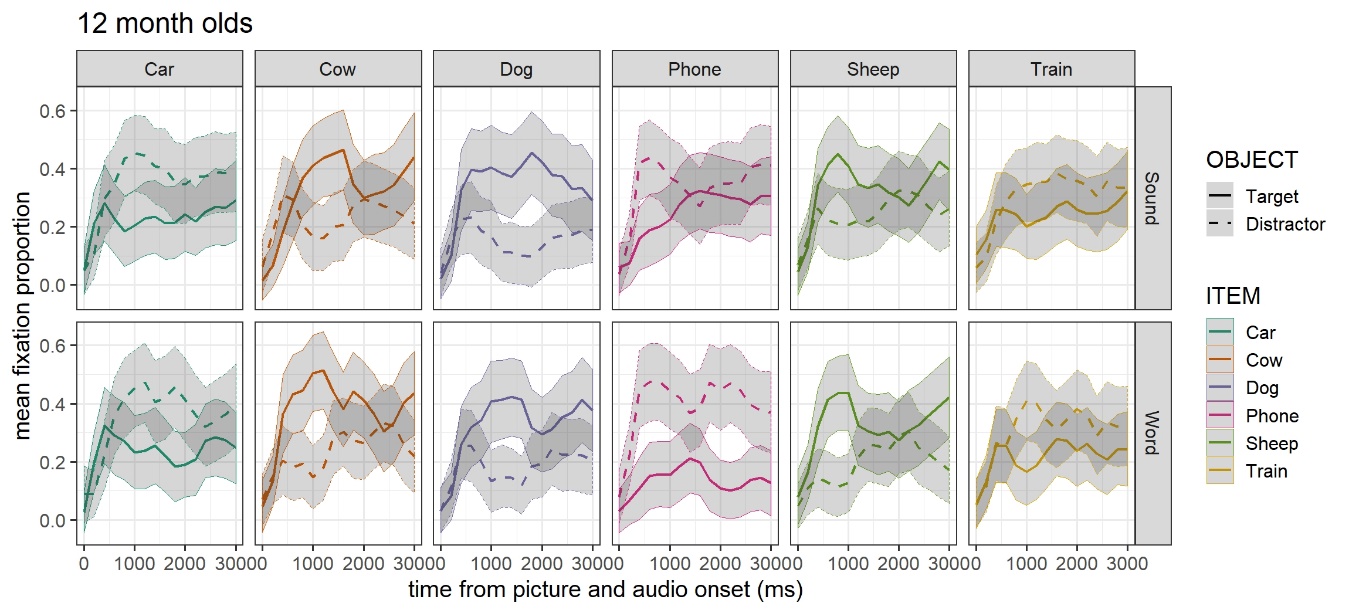


**Figure A3.** Mean fixation proportion (aggregated by participants) as a function of object in the visual context, type of auditory stimulus and individual items in Experiment 2B. Shaded areas around lines represented 95% confidence intervals adjusted for within-subject designs and multiple time windows.

**1.3. Experiment 2C: 18-month-old infants**


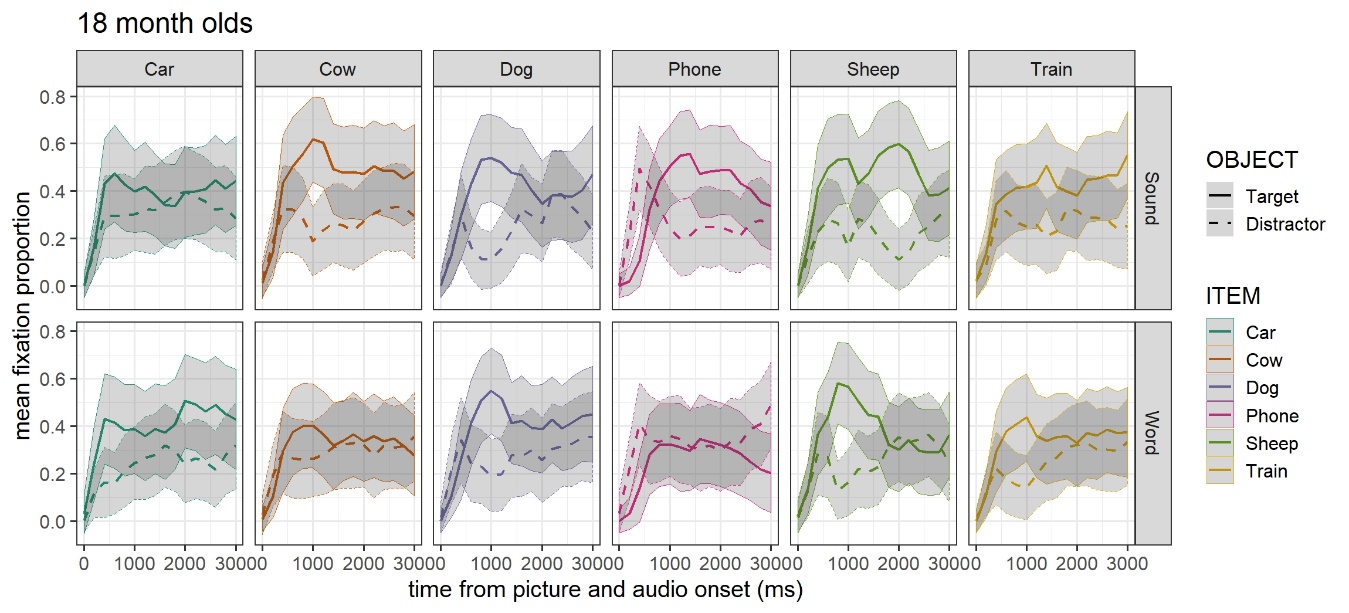


**Figure A4.** Mean fixation proportion (aggregated by participants) as a function of object in the visual context, type of auditory stimulus and individual items in Experiment 2C. Shaded areas around lines represented 95% confidence intervals adjusted for within-subject designs and multiple time windows.

**2. Analysis per item category (for infants’ groups only) and corresponding GCA on animal trials**

The results of the analysis per item revealed that 9- and 12-month-old infants prefer to look at animals(see Figs. A5 and A7), while 18-months-old prefer overall the correct target, but more clearly for animal. We were therefore interested in determining whether infants process words and associated sounds differently within the animal category. The GCA showed that even within the preferred category of animals, words are not more effective at activating conceptual representations at 9- (Fig.A6), 12- (Fig. A8) and 18-month-old (Fig. A10).

**2.1. Experiment 2A: 9-month-old infants**


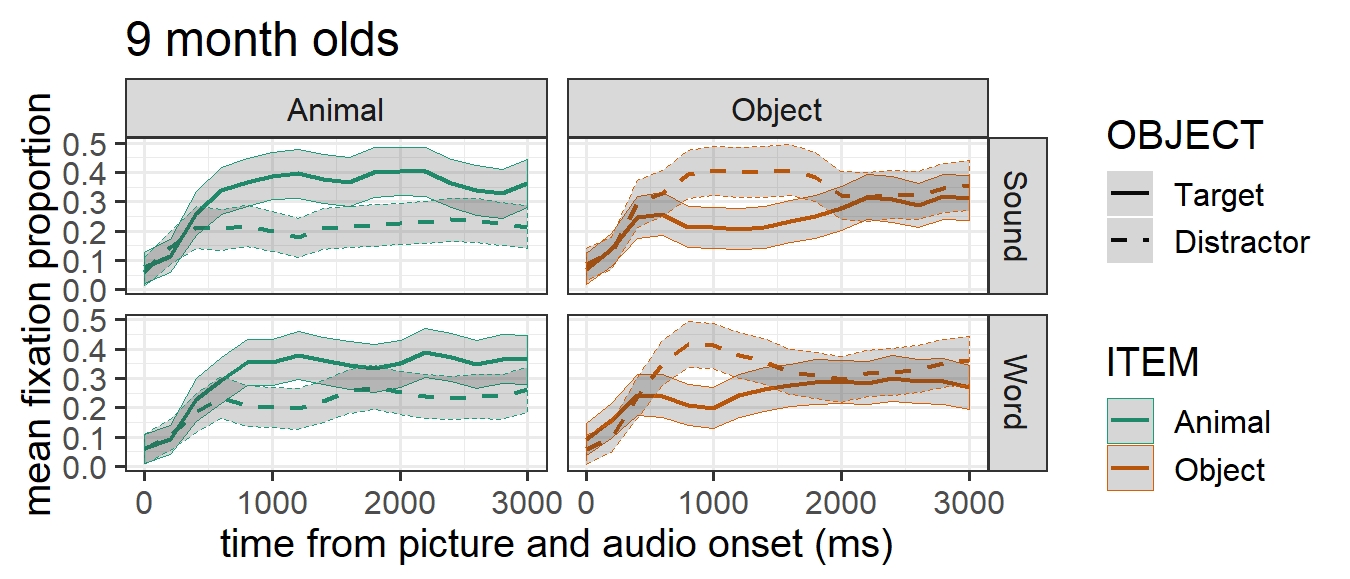


**Figure A5.** Mean fixation proportion (aggregated by participants) as a function of object in the visual context, type of auditory stimulus and items category in Experiment 1B. Shaded areas around lines represented 95% confidence intervals adjusted for within-subject designs and multiple time windows.


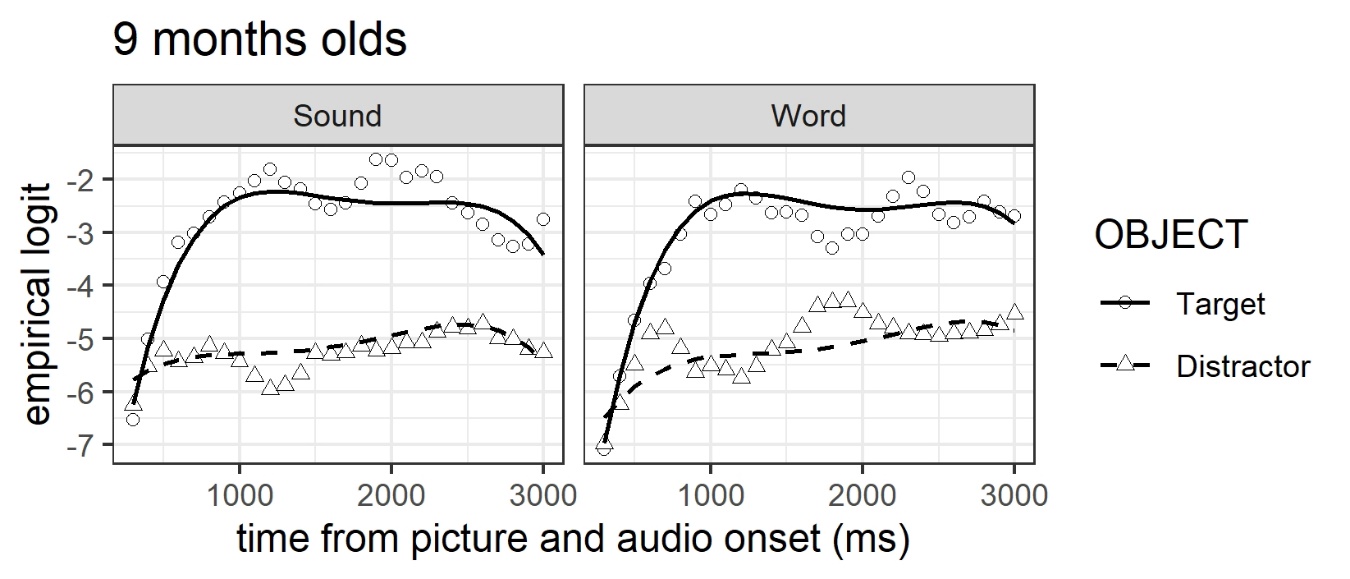


**Figure A6.** GCA model fit (lines) of empirical logit (points) only for animated concepts, as a function of object in the visual context and type of auditory stimulus in Experiment 2A.

The GCA results showed a significant main effect of object type for 9-month-olds (*β* = -1.15, *se* = 0.29, *t* = -3.97), but no effect of words vs. sounds (*β* = 0.03, *se* = 0.21, *t* = 0.17).

**2.2. Experiment 2B: 12-month-old infants**


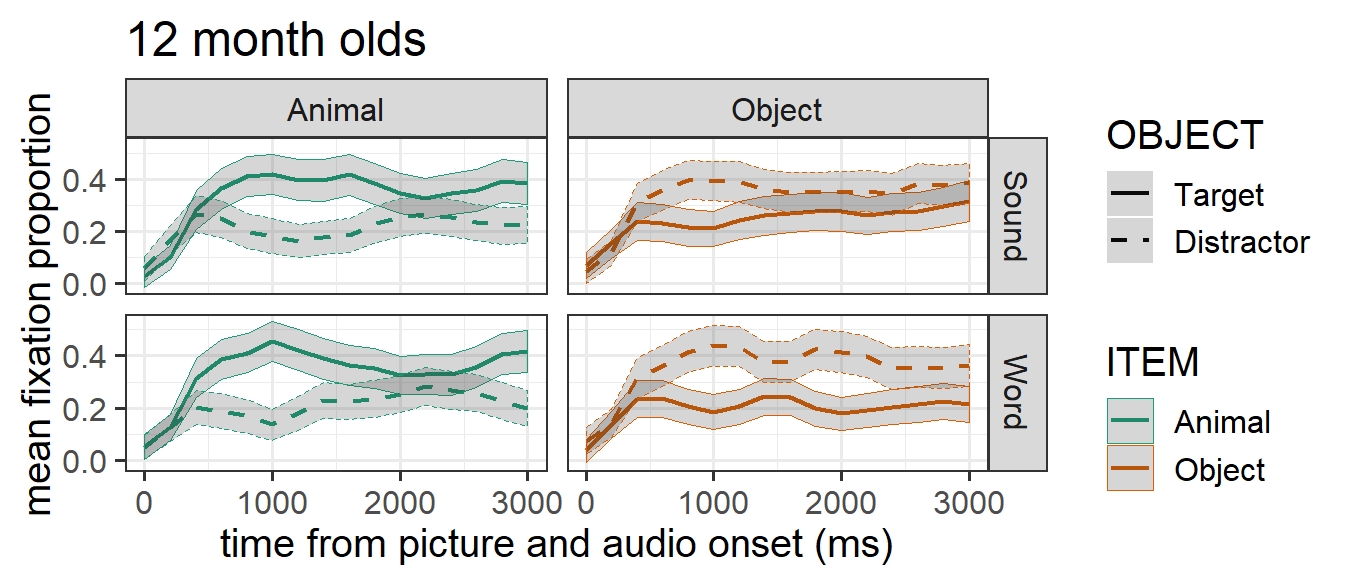


**Figure A7.** Mean fixation proportion (aggregated by participants) as a function of object in the visual context, type of auditory stimulus and items category in Experiment 1B. Shaded areas around lines represented 95% confidence intervals adjusted for within-subject designs and multiple time windows.


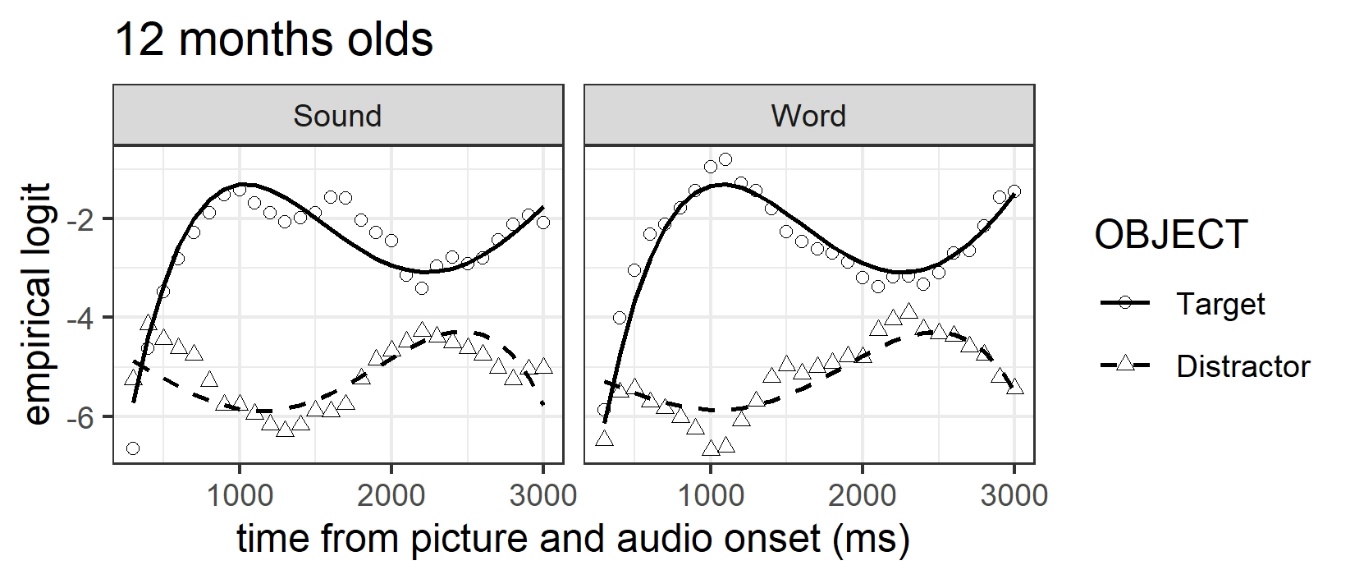


**Figure A8.** GCA model fit (lines) of empirical logit (points) only for animated concepts, as a function of object in the visual context and type of auditory stimulus in Experiment 2B.

The GCA results showed a significant main effect of object type for 12-month-olds (β = -1.33, se = 0.38, t = -3.49), but no effect of words vs. sounds (β = 0.00, se = 0.17, t = 0.04).

**2.3. Experiment 2C: 18-month-old infants**

Eighteen-month-olds (Fig. A9), show a preference for the target over the distractor in the words and associated sounds conditions for both conditions, although this preference is more prominent in the animal relative to the object category. For sake of completeness, we also contrasted (through GCA) the effects of sounds vs. words on the animal category only. Results reveal no reliable difference between these conditions.


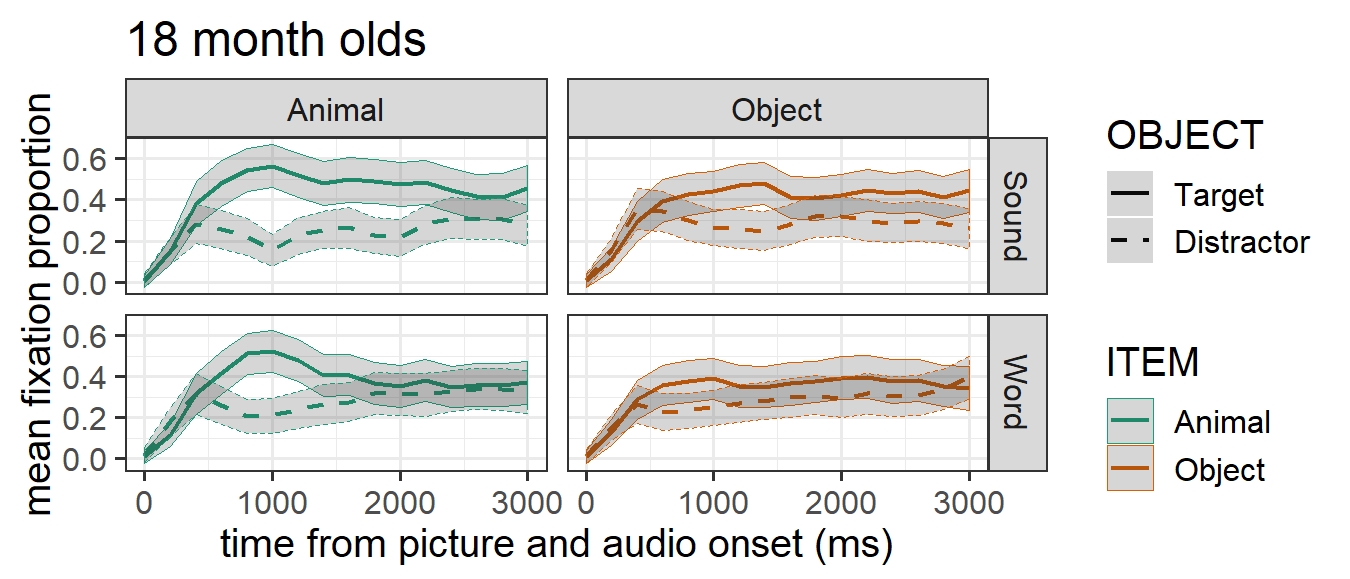


**Figure A9.** Mean fixation proportion (aggregated by participants) as a function of object in the visual context, type of auditory stimulus and items category in Experiment 1B. Shaded areas around lines represented 95% confidence intervals adjusted for within-subject designs and multiple time windows.

**3.3. Experiment 2C: 18-month-old infants**


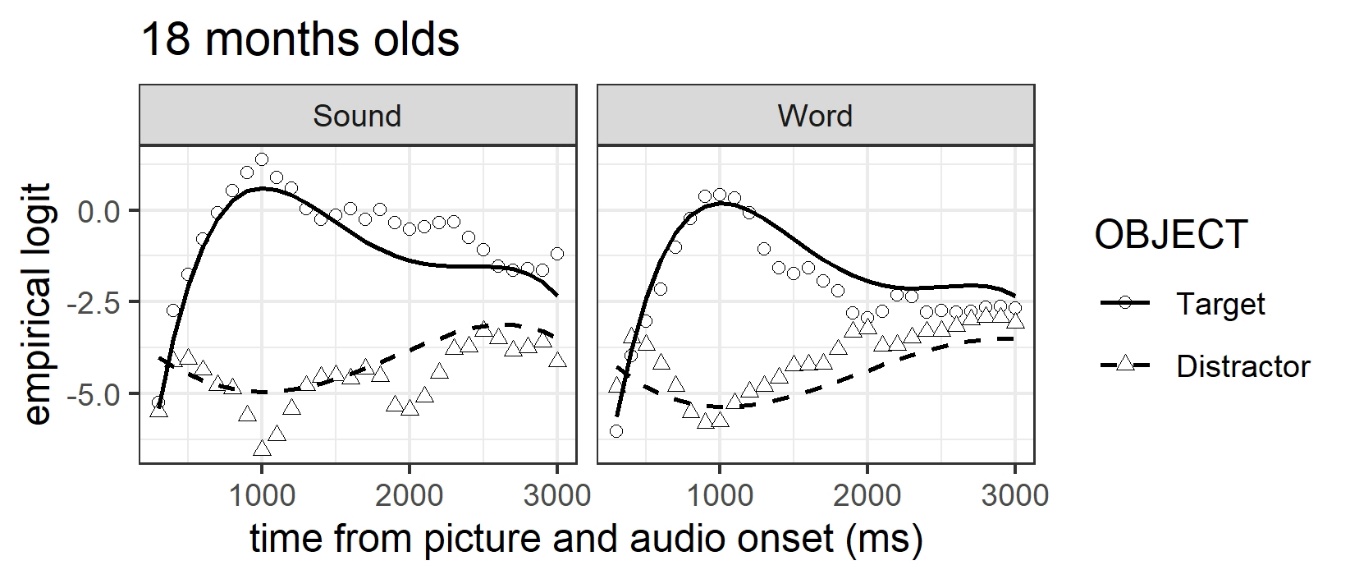


**Figure A10.** GCA model fit (lines) of empirical logit (points) only for animated concepts, as a function of object in the visual context and type of auditory stimulus in Experiment 2C.

The GCA results showed a significant main effect of object type for 18-month-olds (β = -1.47, se = 0.39, t = -3.77), but no effect of words vs. sounds (β = 0.21, se = 0.25, t = 0.85).

**4. Total looking times**

The results of the total looking times further confirm previous analyses showing that only 18-month-old infants looked longer at the target compared to the distractor. These looking times were more prominent in the associated sounds relative to the words condition. On the contrary, in adults longer looking times to the target relative to the distractor were more prominent in the words compared to the associated sounds condition.


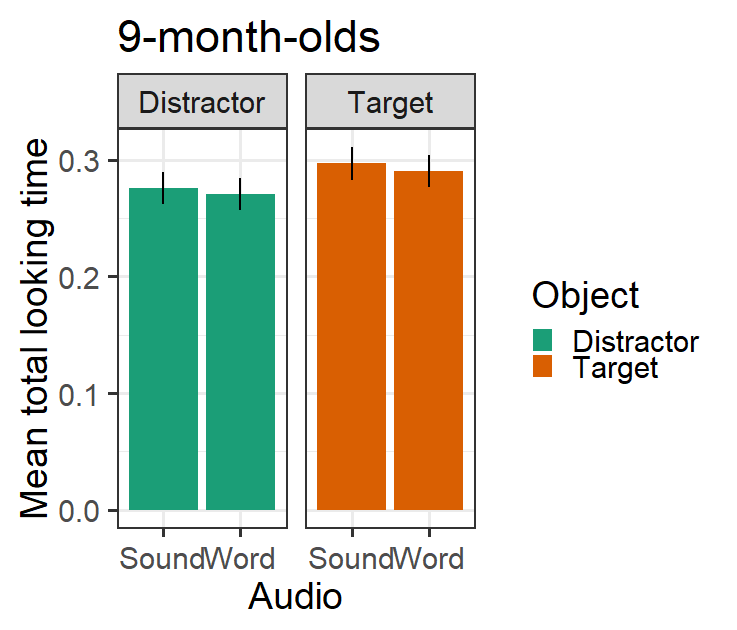

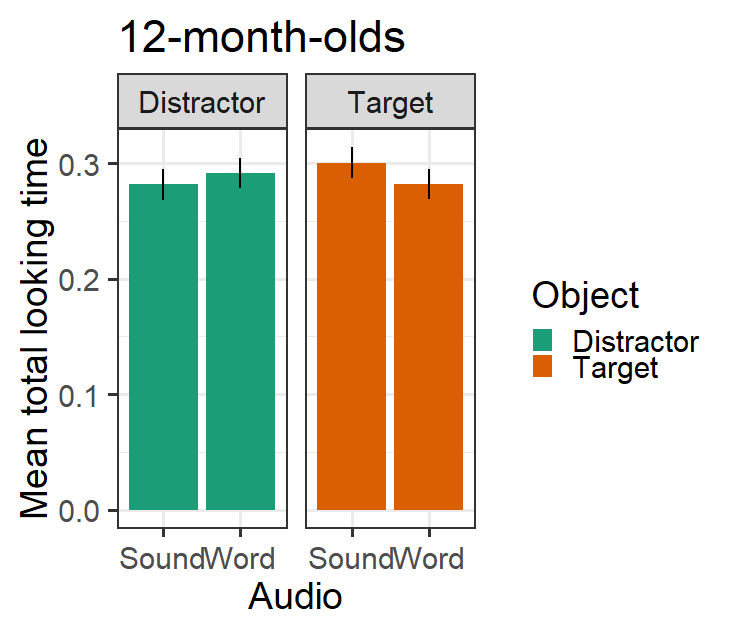

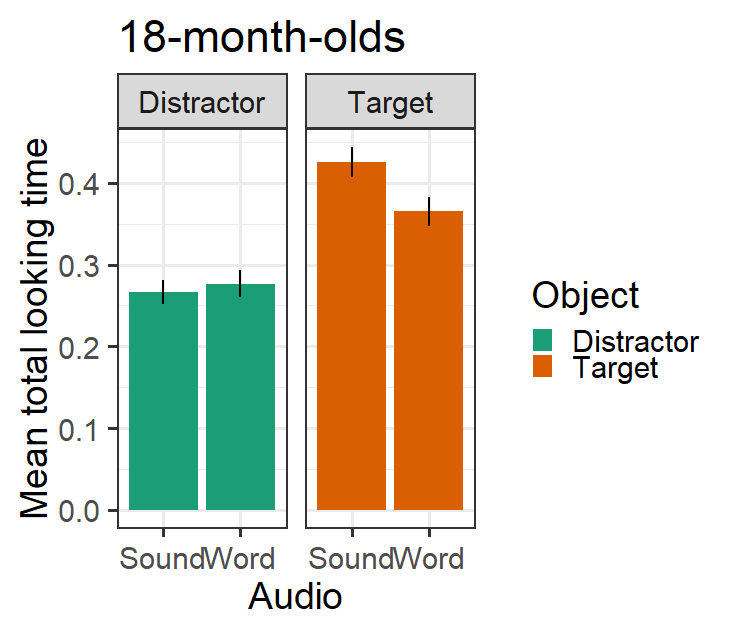

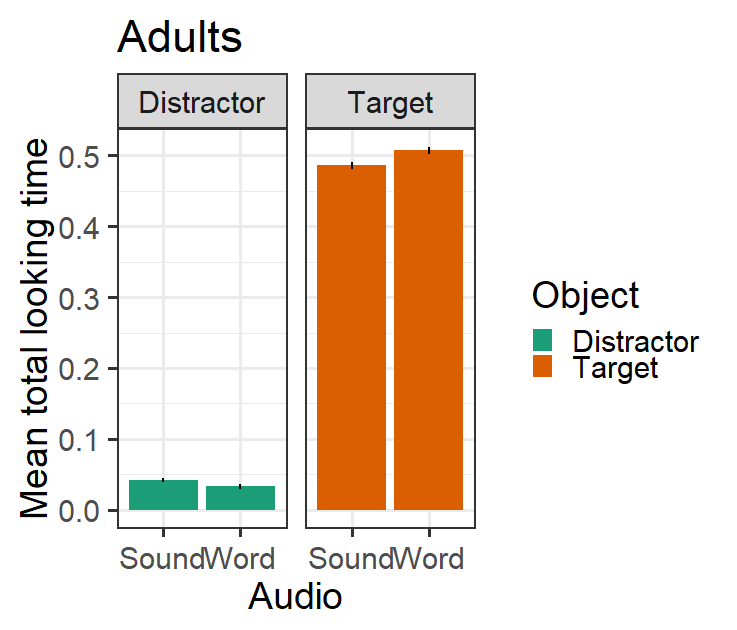

Supplement: S1 Data — (DOCX) [file pone.0233968.s001.docx]
